# Increased expression of long-noncoding RNA ZFAS1 is associated with epithelial-mesenchymal transition of gastric cancer

**DOI:** 10.18632/aging.101048

**Published:** 2016-09-18

**Authors:** Hu Zhou, Fubing Wang, Hao Chen, Qian Tan, Shili Qiu, Shanshan Chen, Wei Jing, Mingxia Yu, Chunzi Liang, Shengwei Ye, Jiancheng Tu

**Affiliations:** ^1^ Department of Clinical Laboratory Medicine and Center for Gene Diagnosis, Zhongnan Hospital of Wuhan University, Wuhan 430071, China; ^2^ Hubei Cancer Hospital, Wuhan 430079, China

**Keywords:** long non-coding RNA, gastric cancer, ZFAS1, epithelial mesenchymal transition, diagnostic marker

## Abstract

LncRNAs play critical roles in gastric cancer (GC). In this study, the expression of fourteen cancer related lncRNAs were investigated in paired tissues of 66 patients with GC, Realtime RT-PCR revealed that ZFAS1 was significantly upregulated. We then examined the expression of ZFAS1 in plasmas derived from 77 GC patients before- and post-operations and 60 healthy individuals, and found that circulating ZFAS1 was also upregulated in GC patients and operation can reduce its presence in plasma. To investigate the potential mechanisms, we compared the expression of ZFAS1 in multiple gastric cell lines and one normal cell line and found that ZFAS1 was up-regulated in GC cell lines. Furthermore, circulating tumor cells (CTC) were simulated by mixing GC cells with peripheral blood. After EpCAM antibody-based cell sorting, we found that the expression of ZFAS1 was positively correlated with EMT property of CTCs. In GC patient tissue samples, we found that Twist was positively correlated with ZFAS1 by immunohistochemical staining. Taken together, our results suggested that ZFAS1 was up-regulated in both tissues and plasmas of GC patients, and may be involved in regulation of EMT in GC progression. Thus, ZFAS1 might serve as a potential diagnostic marker and/or therapeutic target for GC.

## INTRODUCTION

Gastric cancer (GC) is the fourth most commonly diagnosed cancer and the second leading cause of cancer-related deaths worldwide [1, 2]. Approximately 9.5 million new GC cases and 7.2 million deaths are recorded annually [2]. Despite efforts in diagnostic techniques and improvements in chemotherapy and radiotherapy, patients with advanced or metastatic gastric cancer still carry a poor prognosis and the median overall survival (OS) remains less than 1 year [3]. One of the major cause of cancer associated mortality is tumor metastasis, thus, early diagnosis of GC is of particular significance for the prognosis.

Long non-coding RNAs (lncRNAs) are a class of RNA molecules longer than 200 nucleotides with no or limited protein-coding potential [4]. Recently, many studies have revealed that lncRNAs could play critical roles in many biological processes including cellular differentiation, invasion, and metastasis [5-7]. Dysregulation of lncRNA expression has been reported in different types of cancers including lung cancer [8], breast cancer [9], hepatocellular carcinoma [10], and gastric cancer [11]. In addition, circulating lncRNAs has been applied for cancer diagnosis and prognosis [12], and also could be used as potential biomarkers and therapeutic targets for gastric cancer [13].

Epithelial mesenchymal transition (EMT) is a fundamental progress during which epithelial cells transformed into cells with mesenchymal phenotypes. The progress was characterized of losing cellular polarity and adhesion and enhanced cancer cell motility and dissemination [14]. EMT plays a critical role in cancer invasion and metastasis [15]. Previous evidence indicates that lncRNAs may regulate the EMT progression and promote cancer invasion and metastasis [16].

Circulating tumor cells (CTCs) are tumor cells shed into the blood circulation from the primary set and circulate in the bloodstream. CTCs are now considered as a “liquid biopsy” reflecting the progression of cancer [17]. CTCs on the way to potential metastatic sites may act as a diagnostic and prognostic marker for gastric cancer [18]. EMT plays a critical role in cancer invasion and metastasis mediated by CTCs and may promote the progress[15]. Circulating nuclear acids such as miRNAs may act as a surrogate marker for CTCs [19]. LncRNAs also may reflect the CTC levels and act as CTC markers [16].

Here we selected 14 candidate cancer-related lncRNAs (ZFAS1, PVT1, PRNCR1, lncRNA-ATB, HOTAIR, MALAT1, NKILA, TUG1, lnc-MVIH, HULC, lnc-HEIH, UFC1, UCA1 and H19) from previous literatures and a database(LncRNA Disease [20] database) that integrated published lncRNAs and their associated diseases. Among these 14 lncRNAs, we identified ZFAS1 expression is upregulated in tumor tissues and plasmas from GC patients and is positively correlated with clinicopathological features of GC patients. Furthermore, in vitro mechanistic study showed ZFAS1 might be involved in regulation of EMT process of GC cells. More specifically, immunohistochemical analysis of cancer tissue slides revealed ZFSA1 is positively correlated with Twist, a key EMT regulator. These data showed that ZFAS1 may play an important role in gastric cancer invasion and metastasis through regulating EMT and may act as a potential biomarker for diagnosis of gastric cancer, and a marker for EMT and CTCs.

## RESULTS

### ZFAS1 was up-regulated in gastric cancer tissues as compared to the paired normal tissues

**Figure 1:**
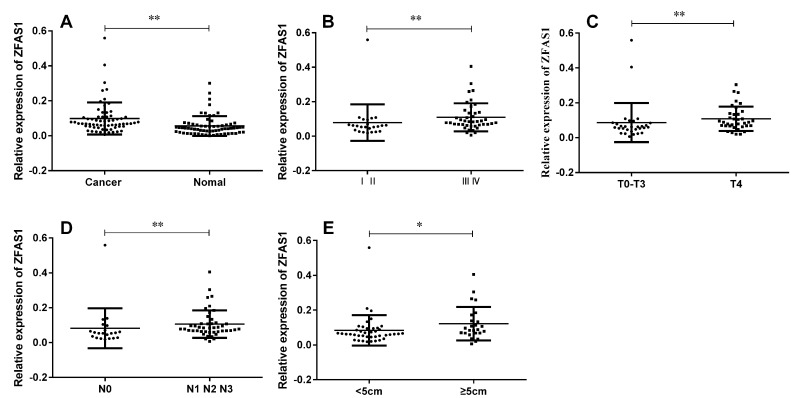
LncRNA ZFAS1 was up-regulated in gastric cancer tissues and was associated with TNM stage, cancer invasion, lymph node metastasis and cancer diameter (**A**) ZFAS1 was highly expressed in GC tissues compared to paired non-tumor tissues (*P* < 0.0001). (**B**) ZFAS1 expression levels in different clinical stage of gastric cancer (*P* = 0.0053). (**C**) Up-regulation of lncRNA ZFAS1 was associated with tumor invasion (*P* = 0.0043). (**D**) Up-regulation of lncRNA ZFAS1 was associated with lymph node metastasis (*P* = 0.0059). (**E**) Up-regulation of lncRNA ZFAS1 was associated with cancer diameter (*P* = 0.0209). The relative expression level was calculated using 2^−ΔCT^ method. **, *P* < 0.01, *, *P* < 0.05. Statistical analysis were conducted by Wilcoxon test (**A**) and Mann-Whitney U test (**B**, **C**, **D** and **E**).

To evaluate the expression of fourteen candidate lncRNAs in GC tissues, we performed realtime RT-PCR to quantify these lncRNAs in GC tissues and the paired normal tissues derived from sixty-six GC patients. The result showed that among these lncRNAs, only ZFAS1 expression level was significantly up-regulated in gastric cancer tissues compared with paired normal tissues, while the expression levels of other lncRNAs did not differ significantly (Fig. 1A). Next, we tested the correlation between the ZFAS1 expression level and the clinicopathological characteristics of the 66 gastric cancer patients, we found that ZFAS1 was highly correlated with TNM stage (Fig. 1B), cancer invasion (Fig. 1C), lymph node metastasis (Fig. 1D) and cancer diameter (Fig. 1E), while there was no significant correlation between ZFAS1 expression level and gender, age, cancer differentiation, or CEA and CA199 levels (summarized in Table 1).

**Table 1:** Correlation between ZFAS1 level in tissues and clinicopathologic of GC

Feature	No. of cases	Mean ± SD	*P*
**Age**			
≥60	34	0.0787 ± 0.0526	0.2621
< 60	32	0.1196 ± 0.1174	
**Gender**			
Male	38	0.0814 ± 0.0668	0.5168
Female	28	0.0676 ± 0.1158	
**Cancer location**			0.9979
Distal	18	0.1008 ± 0.0976	
Middle	33	0.0885 ± 0.0861	
Proximal	35	0.0953 ±0.0998	
**Diameter (cm)**			
≥5	25	0.1220 ± 0.0959	0.0209
< 5	41	0.0842 ± 0.0871	
**Differentiation**			
Well	6	0.0707 ± 0.0443	0.2012
Moderate	18	0.0707 ± 0.0443	
Poor	42	0.1140 ± 0.1079	
**TNM stage**			
0, I and II	24	0.0790 ± 0.1060	0.0053**
III, IV	42	0.1097 ± 0.0817	
**Invasion**			
T0-T3	6	0.0223 ± 0.0273	0.0043**
T4	60	0.0293 ± 0.0249	
**Lymph-node metastasis**			
N0	20	0.0823 ± 0.1145	0.0059**
N1-N3	46	0.1061 ± 0.0792	
**Distant metastasis**			
M0	61	0.0972 ± 0.0930	0.4917
M1	5	0.1144 ± 0.0802	
**CEA**			
Positive	11	0.0855 ± 0.0485	0.8359
Negative	55	0.1011 ± 0.0982	
**CA19-9**			
Positive	15	0.0947 ± 0.0707	0.8886
Negative	51	0.0996 ± 0.0976	
Negative	51	0.0996 ± 0.0976	

### ZFAS1 was highly expressed in the plasmas of preoperative patients than paired postoperative plasmas and the healthy controls

**Figure 2:**
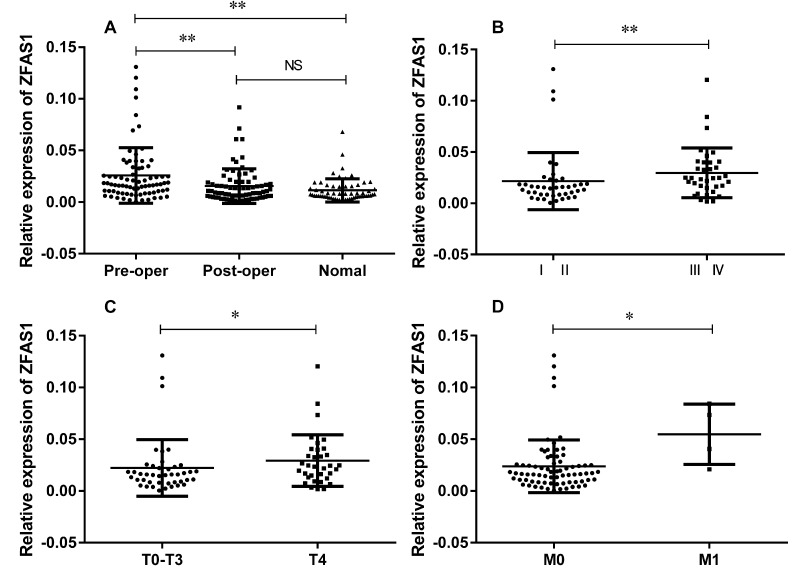
Relative expression level of lncRNA ZFAS1 in 77 paired GC plasmas and 60 healthy controls (**A**) ZFAS1 was highly expressed in GC patients compared to healthy controls (*P* < 0.0001) and patients after surgical treatment (*P* < 0.0001). (**B**-**D**) ZFAS1 was highly expressed in patients with high TNM stage (**B**), depth invasion (**C**), and distant metastasis (**D**). The relative expression level was calculated using 2^−ΔCT^ method. **, *P* < 0.01, *, *P* < 0.05. Statistical analyses were conducted by Wilcoxon test (**A**) and Mann-Whitney U test (**B**, **C**, **D** and **E**).

In addition to expression in tissues, we also examined ZFAS1 expression in seventy-seven paired plasma samples derived from GC patients and sixty plasma samples from healthy controls. There were no significant differences between the GC cases with the healthy controls in the frequency of age distribution (*P* = 0.2114). The mean age was 57.6 in the patient group and 54.9 in the healthy group. In consistent with our findings in tissues, we found that the ZFAS1 expression in preoperative GC plasmas was up-regulated as compared with the paired postoperative ones and the healthy controls (Fig. 2A). Similar to the results in tissues, circulating ZFAS1 level was highly correlated with TNM stage (Fig. 2B), lymph node metastasis (Fig. 2C) and distant metastasis (Fig. 2D), while there was no correlation with other clinicopathologic factors, such as age, gender, cancer differentiation, or CEA and CA199 levels (summarized in Table 2), indicating ZFAS1 might serve as a potential diagnostic maker for GC.

**Table 2:** Correlation between ZFAS1 level in plasma and clinicopathologic of GC

Feature	No. of cases	Mean ± SD	*P*
**Age**			
≥60	37	0.0227 ± 0.0242	0.3841
<60	40	0.0278 ± 0.0282	
**Gender**			
Male	46	0.0258 ± 0.0271	0.9486
Female	31	0.0248 ± 0.0255	
**Cancer location**			
Distal	17	0.0314 ± 0.0298	0.5767
Middle	25	0.0221 ± 0.0307	
Proximal	35	0.0264 ± 0.0227	
**Diameter(cm)**			
≥5	18	0.0318 ± 0.0324	0.3493
<5	59	0.0234 ± 0.0242	
**Differentiation**			
Well	7	0.0214 ± 0.0159	0.135
Moderate	24	0.0185 ± 0.0214	
Poor	46	0.0296 ± 0.0293	
**TNM stage**			
0, I and II	31	0.0216 ± 0.0278	0.0052*
III, IV	36	0.0297 ± 0.0242	
**Invasion**			
T0-T3	43	0.0223 ± 0.0273	0.0285*
T4	34	0.0293 ± 0.0249	
**Lymph-node metastasis**			
N0	44	0.0223 ± 0.0243	0.2937
N1-N3	33	0.0279 ± 0.0279	
**Distant metastasis**			
M0	73	0.0238 ± 0.0254	0.0154*
M1	4	0.0548 ± 0.0292	
**CEA**			
Positive	13	0.0265 ± 0.0229	0.5121
Negative	64	0.0251 ± 0.0271	
**CA19-9**			
Positive	11	0.0283 ± 0.0360	0.4397
Negative	66	0.0249 ± 0.0247	
Negative	66	0.0249 ± 0.0247	

### ZFAS1 was relatively stable in peripheral blood and plasma for conventional detection methods

**Figure 3:**
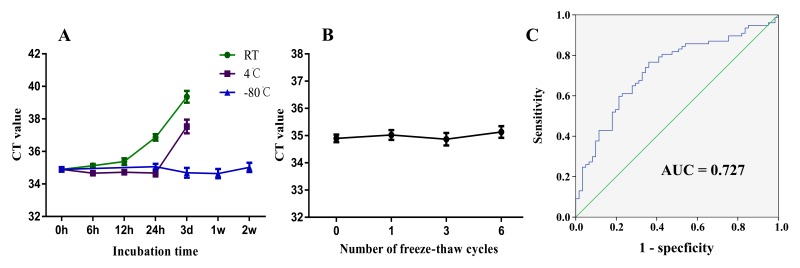
ZFAS1 is suitable for biomarker testing and may serve as a novel biomarker for GC (**A**) The expression level of lncRNA ZFAS1 remained stable when incubated at room temperature for 6h and at 4°C for 12h. ZFAS1 was stable when stored at −80°C. (**B**) ZFAS1 remained relatively stable when treated with freeze-thaw cycles. (**C**) The ROC curve analysis for the diagnostic value of lncRNA ZFAS1 in GC (AUC = 0.727, sensitivity: 0.766; specificity: 0.639).

To investigate the potential application for using ZFAS1 as a potential plasma biomarker for GC, we first tested the stability of ZFAS1 in plasma and the whole blood. The whole blood was aliquoted and incubated in different conditions. Plasma was subjected to six cycles of freezing and thawing to mimic potential clinical detection scenario. Based on realtime RT-PCR analysis, the expression level of ZFAS1 was relatively stable when incubate at room temperature for 6h (*P* = 0.6668), while significant reduction was observed when stored over 12h (*P* = 0.0071). When we stored plasma samples at 4*°*C, we found no statistical differences when the samples were incubated for 12h (*P* = 0.9318) or 24h (*P* = 0.0713), until incubated for 3d (*P* < 0.0001) (Fig. 3A).

Plasma ZFAS1 level was stable when stored at −80°C and the freeze-thaw process had no obvious effects on the stability of ZFAS1 (Fig. 3B). Finally, we tested whether ZFAS1 could be used as a marker of gastric cancer. We used the healthy plasma as a control to produce a ROC curve, which has been established as a standard to determine the value of biomarker. The area under the ROC curve was 0.727 (95% CI=0.642–0.813, *P* < 0.001; Fig. 3C), with a sensitivity of 0.766 and specificity of 0.639. The Youden index was 0.406. Thus, our results provide evidence that plasma ZFAS1 level can be used to distinguish GC patients from controls.

### ZFAS1 was up-regulated in GC cell lines compared in normal gastric cell line

**Figure 4:**
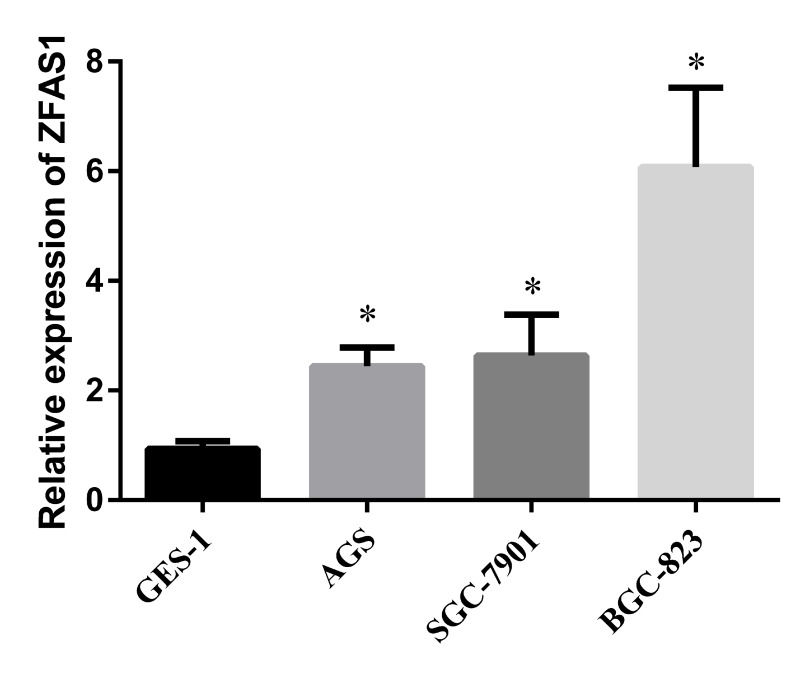
Evaluation of ZFAS1 in three GC cell lines compared to a normal gastric cell line GES-1 The expression level of ZFAS1 in gastric cancer cell lines (AGS, SGC-7901, and BGC-823) were higher than that in human gastric epithelial cell line GES-1, *, *P* < 0.05. The relative expression level was calculated using 2^−ΔΔCt^ method.

To study the cellular and molecular mechanisms underlying ZFAS1 mediated GC progression, we investigated the expression levels of ZFAS1 in three GC cell lines (AGS, SGC-7901 and BGC-823) and one normal cell line GES-1. The result showed that ZFAS1 was highly expressed in GC cell lines than that in normal cell line (*P* < 0.05) (Fig. 4), thus, we can use these cell lines to perform more mechanistic studies.

### ZFAS1 was down-regulated in simulated CTCs compared to un-captured cells and was positively correlated to the mRNA expression levels of mesenchymal markers and negatively correlated to epithelial markers

**Figure 5:**
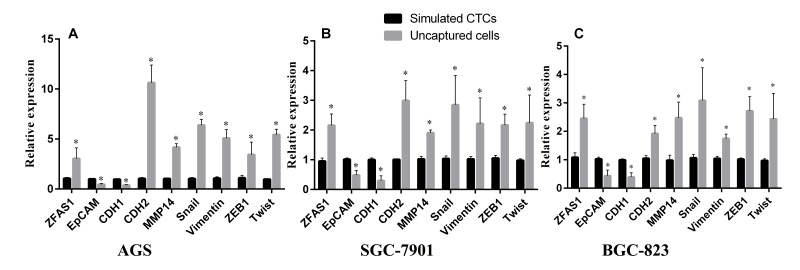
Evaluation of lncRNA ZFAS1 and EMT markers expression level in CTCs simulated by three GC cell lines ZFAS1 level was positively correlated with mesenchymal markers and negatively correlated with epithelial markers in three GC cell lines simulated by AGS (**A**), SGC-7901 (**B**), and BGC-823 (**C**).

EMT and CTCs have been shown to play an important role in gastric cancer progression and metastasis [21]. EpCAM (epithelial cell adhesion molecule) is a transmembrane glycoprotein which is over expressed in numbers of solid tumor cells and is absent from hematologic cells. To explore the potential role of ZFAS1 in CTCs and EMT process, we simulated CTCs by mixing tumor cells with peripheral blood. By using biotinylated epithelial cell adhesion molecule antibody (anti-EpCAM) and streptavidin coated magnetic particles, CTCs were divided into two groups (bind and un-bind groups). The captured cells were the so-called simulated CTCs with high expression of EpCAM, while uncaptured cells express no or low EpCAM. To determine the molecular signatures of two groups of cells, we tested the levels of ZFAS1 and the EMT markers, CDH1, CDH2, Snail, ZEB1*,* Vimentin, MMP14 and Twist by qRT-PCR. We found a decreased expression of the epithelial markers CDH1, EpCAM, and increased expression of the mesenchymal markers CDH2, Vimentin, ZEB1, Snail, MMP14 and Twist in un-captured cells in gastric cancer cell lines AGS, SGC7901 and BGC-823 (Fig. 5). More importantly, expression of ZFAS1 is upregulated in un-captured cells as well, indicating ZFAS1 is enriched in mesenchymal type of cancer cells.

### Correlation between ZFAS1 and Twist in cancer tissues derived from GC patients

**Figure 6:**
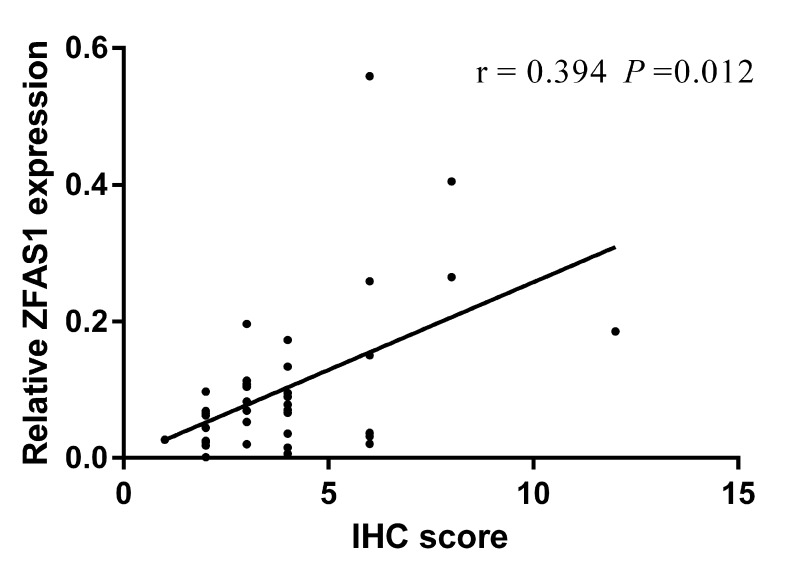
The correlation between ZFAS1 level and Twist ZFAS1 level was positively correlated to the expression level of Twist. The correlation coefficient was 0.394 (*P* < 0.05).

To further investigate the relationship between ZFAS1 and the EMT associated factors, we randomly selected 40 GC tissues to detect the expression level of Twist, a key EMT factor, with immunohistochemical staining. Then, we analyzed the correlation between ZFAS1 and Twist, and found that ZFAS1 expression is positively correlated with Twist. Spearman correlation analysis showed the correlation coefficient was 0.394(*P* < 0.05) (Fig. 6). Thus, we demonstrated, in human cancer tissues, that expression of ZFAS1 is closely correlated with EMT process of GC, which can potentially be targeted for treatment of GC.

## DISCUSSION

LncRNAs play important roles in the development of gastric cancer [22]and can be used as a marker for GC diagnosis [23]. In this study, fourteen lncRNAs were selected from the database and literatures to test their potential roles in GC. We found that ZFAS1 is up-regulated in the GC tissues compared with the paired normal tissues. Furthermore, we found the correlation between the ZFAS1 level and the clinicopathological characteristics of the 66 gastric cancer patients and found that the expression level of ZFAS1 is positively correlated to the TNM stage, cancer invasion, lymph node metastasis and cancer diameter.

Circulating biomarkers including lncRNAs are one of the most promising means of diagnosis for the easy access of serum or plasma, and recent studies have showed that lncRNAs are quite stable in plasma or serum [13, 23], while the precise mechanism is still unclear. A possible explanation is that they could be packaged and protected by several microparticles, such as exsomes [24]. Consistently, our results indicated that lncRNA ZFAS1 was sufficiently stable in plasma. More importantly, we detected the expression level of ZFAS1 in 77 paired plasma samples of GC patients obtained before and after surgery. We found that the expression level of ZFAS1 was higher in preoperative plasmas than postoperative plasmas and the healthy controls. Also plasma ZFAS1 level was associated with TNM stage, cancer invasion, lymph node metastasis and distant metastasis. There exist litter differences in the subgroup analyses between tissue and plasma because some samples were too small and were all used for clinical application. The results indicated that ZFAS1 expression level had a significant predictive value for gastric cancer, and the AUC value for discriminating GC patients from healthy controls was 0.760.

As tumor cells proliferate, they promote angiogenesis and invade into the bloodstream, survive, spread, extravagate, escape into the parenchyma and develop into distant metastasis [25]. CTCs released from primary solid tumors into the vascular circulation are now considered a real-time “liquid biopsy” reflecting the progression of the disease [17]. CTCs invaded into the bloodstream is one of the leading causes for the distant metastasis of cancer [26], and these CTCs may act as a prognostic marker [27].

Recent studies have shown that circulating nuclear acids including miRNAs and lncRNAs may act as a marker for CTCs. Madhavan's [19] study showed that circulating miRNAs can predict the CTC status in patients with metastatic breast cancer. Ortega's [28] study showed that miRNA-21 may act as a good marker for EMT phenotype CTCs. Also, Yuan's [16] study showed that lncRNA-ATB may increase CTC numbers, indicated that lncRNA may also act as a CTC marker.

EMT plays a crucial role in the progress of cancer invasion and metastasis through a variety of mechanisms [15]. Numbers of transcription factors may induce EMT, including zinc finger proteins of the SNAIL superfamily Snail1 and Snail2, zinc finger and E-box binding proteins of the ZEB family ZEB1 and ZEB2, and he TWIST bHLH proteins (TWIST and TWIST2) [29]. These factors may repress the gene transcription by binding to the promoter region of genes involved in cell-cell adhesion such as E-cadherin (CDH1) [30-33], and allow dissociation of cancer cells from the epithelial matrix [14]. Also, the ZEB protein may increase the expression of matrix metallo-proteinases (MMPs) [34] which may promote the invasion and metastasis by degrading the extracellular matrix (ECM) proteins.

Recent studies have shown that EMT markers such as Twist [35] are overexpressed in CTCs, while CTCs expressed low but detectable level of epithelial markers such as keratins, MUC1, EpCAM and/or CDH [36, 37]. Also Lindsay's study [37] showed that patients with Vimentin-positive CTCs has a reduction in OS (overall survival). Angela's study [38] showed that patients with EMT-positive CTCs had a short PFS (perfect forward secrecy).

**Figure 7:**
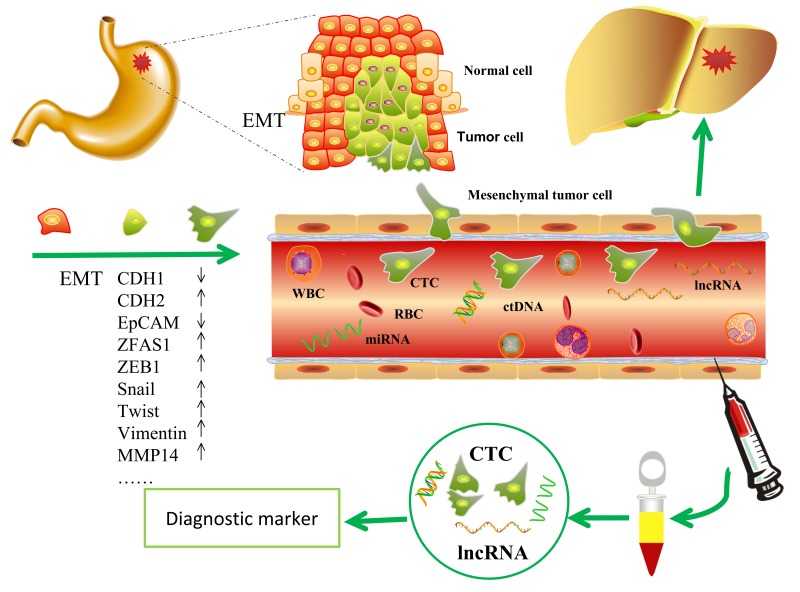
The potential role of lncRNA ZFAS1 in GC ZFAS1contributed to the regulation of EMT in GC progression and thus may serve as a potential diagnostic marker for GC.

During EMT, cells lose their epithelial characteristics and transfer to mesenchymal phenotype [15], change their shape, motility, and adhesion through numerous mechanisms [39], resulting in increased invasive and migratory capability. Thus, elevated numbers of the tumor cells leave the primary parenchyma and enter into the systemic circulations during cancer metastasis [40]. During the progress, several epithelial markers such as EpCAM and CDH1 decreased, while the mesenchymal makers such as CDH2, Snail, ZEB1, Vimentin and Twist increased [14, 15]. EpCAM is up-regulated in numerous solid tumor cells and is absent from hematologic cells [41], so that EpCAM is used as a surface marker to capture circulating tumor cells [42, 43]. The epithelial surface markers EpCAM and CDH1 may down-regulated after EMT [44, 45]. In this study, three representative cell lines of GC were chosen and ZFAS1 was investigated with a non-tumor gastric cell line as control. The data showed that ZFAS1 was highly expressed in GC cell lines (AGS, SGC7901 and BGC-823) compared in non-tumor gastric cell line GES-1. To investigate the mechanism, we simulated CTCs by mixing tumor cells into peripheral blood. Cells were divided into two groups by biotinylated EpCAM antibody and streptavidin coated magnetic particles. Cells captured by magnetic particles expressed a high level of EpCAM. We compared the expression level of ZFAS1 and EMT markers in the two groups, we found that the expression level of ZFAS1 was positively related with the mesenchymal markers CDH2, Vimentin, ZEB1, Snail, MMP14 and Twist, and was negatively related with the epithelial marker CDH1 and EpCAM. These results indicated that ZFAS1 may play an important role in the progress of EMT, which may induce the metastasis of gastric cancer, and ZFAS1 may act as a diagnostic marker for gastric cancer and a mesenchymal marker for EMT and CTCs (Fig.7).

A previous study [46] showed that ZFAS1 is down-regulated in breast tumors compared with normal tissues and the silencing of ZFAS1 in mammary epithelial cell line increased cellular proliferation and differentiation, indicated that ZFAS1 may act as a tumor suppressor gene and the mechanisms were unknown. While another study [47] showed that ZFAS1 transcripts are highly expressed in most HCC tissues compared with the paired non-tumor tissues and the upregulation of ZFAS1 promoted the metastasis through a miR-150 dependent manner. ZFAS1 act as a miR-150 sponge and inhibited miR-150, leading to the activating of ZEB1, MMP14 and MMP16. Recent study showed that ZFAS1 may destabilize p53 and interact with CDK1/cyclin B1 complex leading to cell cycle progression and inhibition of apoptosis [48]. Our results showed that ZFAS1 is up-regulated in GC tissues compared with the paired normal tissues, and plasma ZFAS1 level is down-regulated after operation, and the expression level of ZFAS1 is positively related with mesenchymal markers and negatively related with epithelial markers. Thus, it is possible that ZFAS1 plays different roles in different type of cancers, and the mechanism requires further investigation.

## MATERIALS AND METHODS

### Tissue and plasma samples

Sixty-six fresh paired human gastric tumor tissues and seventy-seven paired plasma (preoperative and postoperative) samples were obtained from patients with gastric cancer undergoing surgery in Zhongnan Hospital of Wuhan University between June 2014 and June 2015. The postoperative ones were collected two weeks after surgery. All patients had been pathological diagnosed as GC and none of them had previously undergone radiotherapy or chemotherapy treatment. Sixty control samples were collected from healthy volunteers without cancerous diseases. All Blood samples obtained from each donor were placed in the EDTA-anticoagulant tube within 1h. The plasma were separated by centrifugation at 1000 g for 10min at 4°C, followed by a 15min high-speed centrifugation at 10000 g at 4°C to completely remove cell debris. Collected clinical data contained information about tumor localization, TNM stage according to the American Joint Committee on Cancer. Tissues and plasmas were stored at −80°C until use. The study was approved by the Institutional Ethics Committee of Zhongnan Hospital of Wuhan University (approval number: 2013059).

### Cell culture

All cell lines were cultured at 37°C in a 5% CO_2_ incubator. Cell lines GES-1, BGC-823, AGS, and SGC-7901 were purchased from CCTCC (China Center for Type Culture Collection). GES-1 and SGC-7901 were maintained in DMEM (Gibco, Invitrogen, USA) supplemented with 10% fetal bovine (Gibco, Invitrogen, USA). AGS and BGC-823 were maintained in RPMI medium 1640 (Gibco, Invitrogen, USA) supplemented with 10% fetal bovine (Gibco, Invitrogen, USA).

### Cell sorting

AGS, SGC-7901 and BGC-823 were used as the target and the cells were counted and tested for viability before experiments. Cells were first dissociated by PBS containing 0.02% EDTA, and were suspended in 1ml peripheral blood. Then the target cells (1×10^6^) were washed and then incubated with 5μl Human EpCAM/TROP-1 Biotinylated Antibody (CAA32870, R&D Systems China Co. Ltd., Shanghai, China) in 4°C on an orbital shaker for 60min. After incubation, the cells were washed three times and were incubated with 30μl streptavidin coated magnetic particles (Dynabeads M-280 Streptavidin, 11205D, Invitrogen, USA) on an orbital shaker in 37°C for 30min. The bounded cells were separated by the beads and were used for RNA isolation.

### RNA isolation and cDNA synthesis

Total RNA was isolated from the tissues and cells using TRIzol reagent (Invitrogen, United States of America (USA)) according to the manufacturer's instruction. The plasma total RNA was extracted from 250μl of plasma using a Blood Total RNA Isolation Kit (RP4001, BioTeke, Beijing, China) and eluted in 30μl of pre-heated (75°C) elution according to the manufacturer's instruction. The concentration of RNA was measured by Nanodrop 2000 spectrophotometer (Thermo Scientific Inc., USA). cDNA was synthesized using the PrimeScript™ RT reagent Kit with gDNA Eraser (RR047A, Takara, Dalian, China). Reverse transcription conditions were as follows: 42°C for 2 min, and then 37°C for 15 min, 85°C for 5s, followed by storage at 4°C.

### Quantitative real-time PCR (qRT-PCR) assay

Quantitative real-time PCR assay was performed with SYBR^®^ Premix Ex Taq™ II (RR820A, Takara, Dalian, China) on the Bio-Rad CFX96 (Bio-Rad Laboratories, Inc. USA) following manufacturer's instructions. Primer sequences for amplification were designed by Primer 3.0 and the sequences were listed in Supplementary Table 1. The Glyceraldehyde-3-phosphate dehydrogenase (GAPDH) was used as the endogenous control and was amplified simultaneously with target genes.

The PCR reactions were performed in a volume of 20μl (10μl of SYBR green mix, 0.8μl 10μmol sense, 0.8μl 10μmol anti-sense, and 6.4μl water and 2μl cDNA). The cycling program was set for initial hold at 95°C for 30s, followed by 40 cycles of denaturation at 95°C for 5s, annealing at 63.3°C for 30s and extension at 72°C for 30s. All experiments were carried out in duplicate for each data point. Relative gene expression level (the amount of target, normalized to endogenous control gene) was calculated using 2^−ΔCt^ method.

### Immunohistochemical staining

Immunohistochemical staining was used to detect the expression of Twist. The anti-Twist antibody (ab50581, Abcam, England) was diluted 1:300 for the detection. The staining score was based on the staining intensity and the percentage of the positive cells. The staining intensity was scored as 0 (negative), 1 (very weak), 2 (weak), 3 (medium) and 4 (strong). The extent of staining was scored as 0, 0-10%; 1, 10%-30%; 2, 30%-50%; 3, 50%-75%; and 4, >75% according to the percentage of positive-staining cells. The expression of Twist was scored as the sum of the two parts. Slides with a total score over 3 were defined as the positive expression.

### Statistical analyses

Data were presented as mean±SD deviation. Data analyses were performed with Prism6 (GraphPad software, La Jolla, CA) or SPSS version 21.0 software (SPSS, Inc., Chicago, IL). For comparisons, Student *t* test (two tailed), one-way analyses of variance (ANOVA), and Mann-Whitney U test were performed. Statistical differences were set at *P < 0.05, **P < 0.01. *P* < 0.05 was considered statistically significant.

## CONCLUSION

Taken together, the expression of ZFAS1 was significantly increased in GC tumor tissues and plasma, and may decrease after surgery. ZFAS1 may act as a marker for gastric cancer diagnosis, and a marker for EMT and CTCs. ZFAS1 may promote cancer invasion and metastasis by regulating EMT and CTCs. However, the molecular mechanisms still require further investigation.

## SUPPLEMENTARY MATERIAL TABLE AND FIGURE


