# Supplementary material for: Increased expression of long-noncoding RNA ZFAS1 is associated with epithelial-mesenchymal transition of gastric cancer
Source: Aging (Albany NY). 2016 Sep 18;8(9):2023–35. doi: 10.18632/aging.101048 (PMC5076450; doi:10.18632/aging.101048)
Supplement: Supplementary file 1 [file aging-08-2023-s001.pdf]

SUPPLEMENTARY MATERIAL

Table S1. List of primers used in this paper.

Primer name	Sequence (5′-3′)
ZFAS1-F	ACGTGCAGACATCTACAACCT
ZFAS1-R	TACTTCCAACACCCGCAT
PVT1-F	GGGGAATAACGCTGGTGGAA
PVT1-R	CCCATGGACATCCAAGCTGT
PRNCR1-F	CAGCAGCCGATATGATGGTATT
PRNCR1-R	GGTTCTGTTGTCTGGTGATGG
lncATB-F	TCTGGCTGAGGCTGGTTGAC
lncATB-R	ATCTCTGGGTGCTGGTGAAGG
HOTAIR-F	CAGTGGGGAACTCTGACTCG
HOTAIR-R	GTGCCTGGTGCTCTCTTACC
MALAT-F	AAAGCAAGGTCTCCCCACAAG
MALAT-R	GGTCTGTGCTAGATCAAAAGGCA
NKILA-F	AACCAAACCTACCCACAACG
NKILA-R	ACCACTAAGTCAATCCCAGGTG
H19-F	GGGTCTGTTTCTTTACTTCCTCCAC
H19-R	GATGTTGGGCTGATGAGGTCTGG
TUG1-F	ACAACGACTGAGCAAGCACTA
TUG1-R	GGAGGCACAGGACATAATTCACT
GAPDH-F	GGTCTCCTCTGACTTCAACA
GAPDH-R	GTGAGGGTCTCTCTCTTCCT
lncMVIH-F	AATTTTGCACATCTGAACAGCC
lncMVIH-R	TTCAAAATCCCACTACGCCCA
HULC-F	CAGGAACTCTGATCGTGGACATT
HULC-R	CTTGCTTGATGCTTTGGTCTGTTT

HEIH-F	TGGTGGCTTAGGCTTAATCTCAG
HEIH-R	GGTCTCAGTCAGTCATCATTCTCA
UFC1-F	GCTACCCTCTCCCAGTCCTTA
UFC1-R	CTGACCTCCAACTCCAACGAA
UCA1-F	CTCGGCTTAGTGGCTGAAGAC
UCA1-R	TGGTCCATTGAGGCTGTAGAGT
MMP14-F	TGCCTGCGTCCATCAACA
MMP14-R	CCCAATGCTTGTCTCCTTTGAAG
ZEB1-F	CAGCCAAATGGAAATCAGGATGAA
ZEB1-R	GGCGGTGTAGAATCAGAGTCA
Twist-F	GTCCGCAGTCTTACGAGGAG
Twist-R	TGGAGGACCTGGTAGAGGAA
CDH1-F	AGACCAAGTGACCACCTTAGAG
CDH1-R	GAGCAGCAGAATCAGAATTAGCA
CDH2-F	CATCATCATCCTGCTTATCCTTGT
CDH2-R	TTCTCCTCCACCTTCTTCATCAT
Snail-F	GCCTAGCGAGTGGTTCTTCT
Snail-R	TGCTGGAAGGTAAACTCTGGATT
Vimentin-F	GATTTCTCTGCCTCTTCCAAACTT
Vimentin-R	TTCGTTGATAACCTGTCCATCTCT
EpCAM-F	GTGGTTGTGGTGATAGCAGTTG
EpCAM-R	CCATCTCCTTTATCTCAGCCTTCT

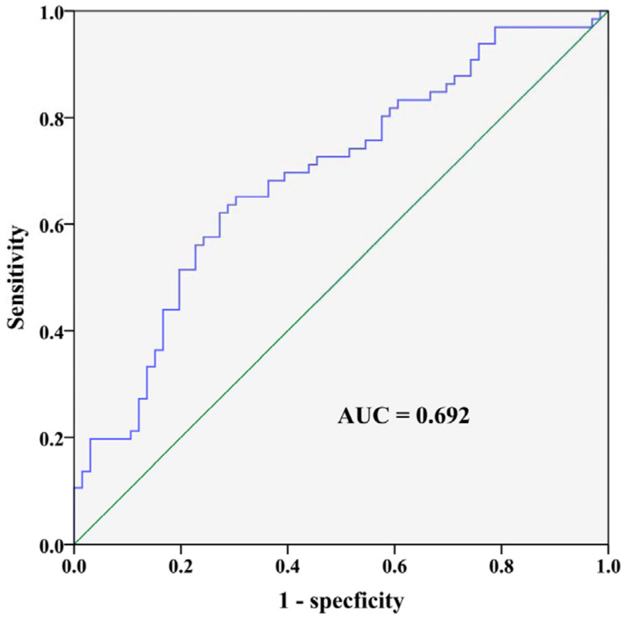

Figure S1. **F**ROC curve for tissue. The ROC curve analysis for the diagnostic value of lncRNA ZFAS1 in tissue of GC (AUC =0.692, sensitivity: 0.653, specificity: 0.696).
